# Zika virus infection in immunocompetent pregnant mice causes fetal damage and placental pathology in the absence of fetal infection

**DOI:** 10.1371/journal.ppat.1006994

**Published:** 2018-04-10

**Authors:** Frank M. Szaba, Michael Tighe, Lawrence W. Kummer, Kathleen G. Lanzer, Jerrold M. Ward, Paula Lanthier, In-Jeong Kim, Atsuo Kuki, Marcia A. Blackman, Stephen J. Thomas, Jr-Shiuan Lin

**Affiliations:** 1 Trudeau Institute, Saranac Lake, New York, United States of America; 2 Global VetPathology, Montgomery Village, Maryland, United States of America; 3 Upstate Medical University, State University of New York, Syracuse, New York, United States of America; University of Pittsburgh, UNITED STATES

## Abstract

Zika virus (ZIKV) infection during human pregnancy may cause diverse and serious congenital defects in the developing fetus. Previous efforts to generate animal models of human ZIKV infection and clinical symptoms often involved manipulating mice to impair their Type I interferon (IFN) signaling, thereby allowing enhanced infection and vertical transmission of virus to the embryo. Here, we show that even pregnant mice competent to generate Type I IFN responses that can limit ZIKV infection nonetheless develop profound placental pathology and high frequency of fetal demise. We consistently found that maternal ZIKV exposure led to placental pathology and that ZIKV RNA levels measured in maternal, placental or embryonic tissues were not predictive of the pathological effects seen in the embryos. Placental pathology included trophoblast hyperplasia in the labyrinth, trophoblast giant cell necrosis in the junctional zone, and loss of embryonic vessels. Our findings suggest that, in this context of limited infection, placental pathology rather than embryonic/fetal viral infection may be a stronger contributor to adverse pregnancy outcomes in mice. Our finding demonstrates that in immunocompetent mice, direct viral infection of the embryo is not essential for fetal demise. Our immunologically unmanipulated pregnancy mouse model provides a consistent and easily measurable congenital abnormality readout to assess fetal outcome, and may serve as an additional model to test prophylactic and therapeutic interventions to protect the fetus during pregnancy, and for studying the mechanisms of ZIKV congenital immunopathogenesis.

## Author summary

Zika virus (ZIKV) infection during human pregnancy may cause severe congenital abnormalities and fetal death. There is currently no licensed vaccine or anti-ZIKV therapeutic to prevent or treat infection and/or disease. To generate an animal model that mimics human ZIKV infections, others have manipulated mice to impair their innate immunity, which allows ZIKV to develop high levels of infection. Here, we present an experimental model using immunologically unmanipulated pregnant mice and show that even limited maternal ZIKV infection nonetheless resulted in profound placental pathology and high frequency of fetal demise. However, neither viral RNA level in the dam, placenta or embryo reliably predicted fetal abnormalities. Our studies suggest that, in this model, placental pathology including trophoblast hyperplasia, focal regions of necrosis, and loss of embryonic blood vessels in the placenta likely promote adverse fetal outcomes. This immunocompetent pregnant mouse model provides clear *in vivo* phenotypic readouts (e.g. embryonic viability and gross uterine and embryonic morphology) to assess the potential for clinical benefit of candidate vaccines and therapeutics in a model not dependent on vertical transmission of virus.

## Introduction

Zika virus (ZIKV) is a mosquito-borne virus belonging to the genus Flaviviridae and is closely related to several other arboviruses, including dengue viruses (DENV), yellow fever virus (YFV), West Nile virus (WNV), Japanese encephalitis virus (JEV) and tick-borne encephalitis virus (TBEV) [1]. ZIKV infection is often asymptomatic or presents as a mild, self-limiting febrile illness accompanied by rash, malaise, conjunctivitis, or muscle and joint pains [1–3]. The epidemic in the Americas first noted in 2015 was temporally related to the occurrence of thousands of fetal abnormalities during pregnancy [1–4], and the causal relationship between ZIKV infection and adverse pregnancy outcomes (e.g. congenital ZIKV syndrome, CZS) has since been established in both humans and following experimental infection of pregnant mice and non-human primates [4–13]. CZS encompasses a number of potential abnormalities to include intrauterine growth restriction (IUGR), fetal demise leading to spontaneous abortion, stillbirth, microcephaly, ocular disorders, and developmental abnormalities yet to be fully characterized [14–17]. Because of the devastating consequences of CZS, vaccine and drug countermeasures are being developed at a rapid pace, and the initial focus is to prevent infection and disease in women of child bearing age.

Pre-clinical mouse pregnancy models have been developed to examine the consequences of ZIKV infection on the developing embryo/fetus [6, 7, 18–23]. Mouse models of ZIKV infection are constrained by the virus’s inability to replicate and cause overt clinical symptoms and disease manifestations in immunocompetent mice. It has been demonstrated that the murine innate Type I interferon (IFN) response abrogates ZIKV infection [22–24]. To overcome this innate response, investigators have employed immunocompromised mice genetically lacking Type I IFN signaling or wild-type mice treated with antibodies to block Type I IFN signals [6, 7, 25–27]. Indeed, these models recapitulate several disease manifestations of ZIKV infection observed in humans; however, the utilization of immunocompromised mice prevents the study of potential beneficial or detrimental impact of host immunity on ZIKV disease pathogenesis. Experimental conditions using immunocompetent pregnant mice which have been explored to date include inoculating mice with extraordinarily high doses of virus, or via specialized techniques or surgeries to directly deliver the virus to the maternal-fetal interface (e.g. vaginal exposure, intrauterine infection) [7–10, 18–20]. These studies, too, have had some success. By these manipulations, vertical transmission of ZIKV from the mother to the fetus has been demonstrated in mice [6, 7, 18, 19], despite the fundamental differences of placental structures in humans and mice. These studies showed that fetal damage coincides with ZIKV disseminated to the placenta and embryo. However, these settings created high and sustained viral titers in the circulation or in the proximity of the placenta [6, 7, 19], opening the possibility that the transplacental transmission and fetal damage may have been driven by unusually high maternal viral loads which would not have been achieved in an immunocompetent host.

CZS in human newborns has been reported with asymptomatic maternal ZIKV infection [4, 28], suggesting that maternal innate immune responses may suffice to prevent or significantly attenuate illness, yet not suffice to protect the fetus from infection or poor clinical outcome. Little is known about the consequences of ZIKV infection during pregnancy beyond those resulting directly from vigorous ZIKV infection and vertical transmission. ZIKV infection can also damage the placenta leading to placental insufficiency and pathology [6, 19], introducing the question of whether CZS is caused by direct viral effects on the fetus, secondary effects resulting from primary placental damage, or both.

The impact of ZIKV infection on developing embryos has been shown to vary with the gestational stage [14, 15, 29]. Infection during early pregnancy appears to pose the highest risk of fetal damage in both humans and mouse models [4–6, 19, 20, 28–30]. Growing epidemiological data also indicate the risk of adverse fetal outcome when maternal infection occurs at second and third trimesters [4, 31, 32]. Animal models suggest that early stage infection prior to completion of placentation often results in higher rates of viral transmission and more severe fetal outcomes than later stage infection when the placenta is fully functional [6, 19, 20, 29]. It is conceivable that the window of susceptibility and the impact of ZIKV infection on fetal outcome may depend on gestational stage-associated changes at the maternal-fetal interface.

In this study, we utilized immunologically unmanipulated wild-type C57BL/6J mice, which therefore developed limited ZIKV infection. We found that even pregnant mice competent to generate intact immunity, including type I IFN signaling, nonetheless developed placental pathology and profound fetal abnormalities, including IUGR, embryonic malformation and high frequency of fetal demise. The impact on the developing embryo was caused by live infectious virus and was dependent on the dose and timing of viral administration during pregnancy. However, because viral replication was limited in the immunocompetent mice, vertical transmission was rare and, consequently, fetal abnormality did not reliably associate with the presence of ZIKV in maternal tissues, placenta or embryo. Our results reveal that adverse fetal outcome in the absence of vertical transmission was likely associated with placental pathology, including trophoblast hyperplasia in close proximity to embryonic blood vessels in the labyrinth and trophoblast giant cell necrosis in the junctional zone. Our findings indicate that, in this model, placental pathology leading to placental insufficiency rather than viral transmission can lead to profound adverse fetal outcome. Overall, our findings suggest the importance of a ZIKV pregnancy model that allows detection of placental pathology and fetal abnormality even with very low levels of overall viral infection, in order to test for interventions that can provide protective benefits against the full range of deleterious placental and fetal outcomes beyond merely preventing vertical transmission.

## Results

### Intravenous ZIKV infection of pregnant immunocompetent wild-type C57BL/6J mice results in fetal demise

**Fig 1:**
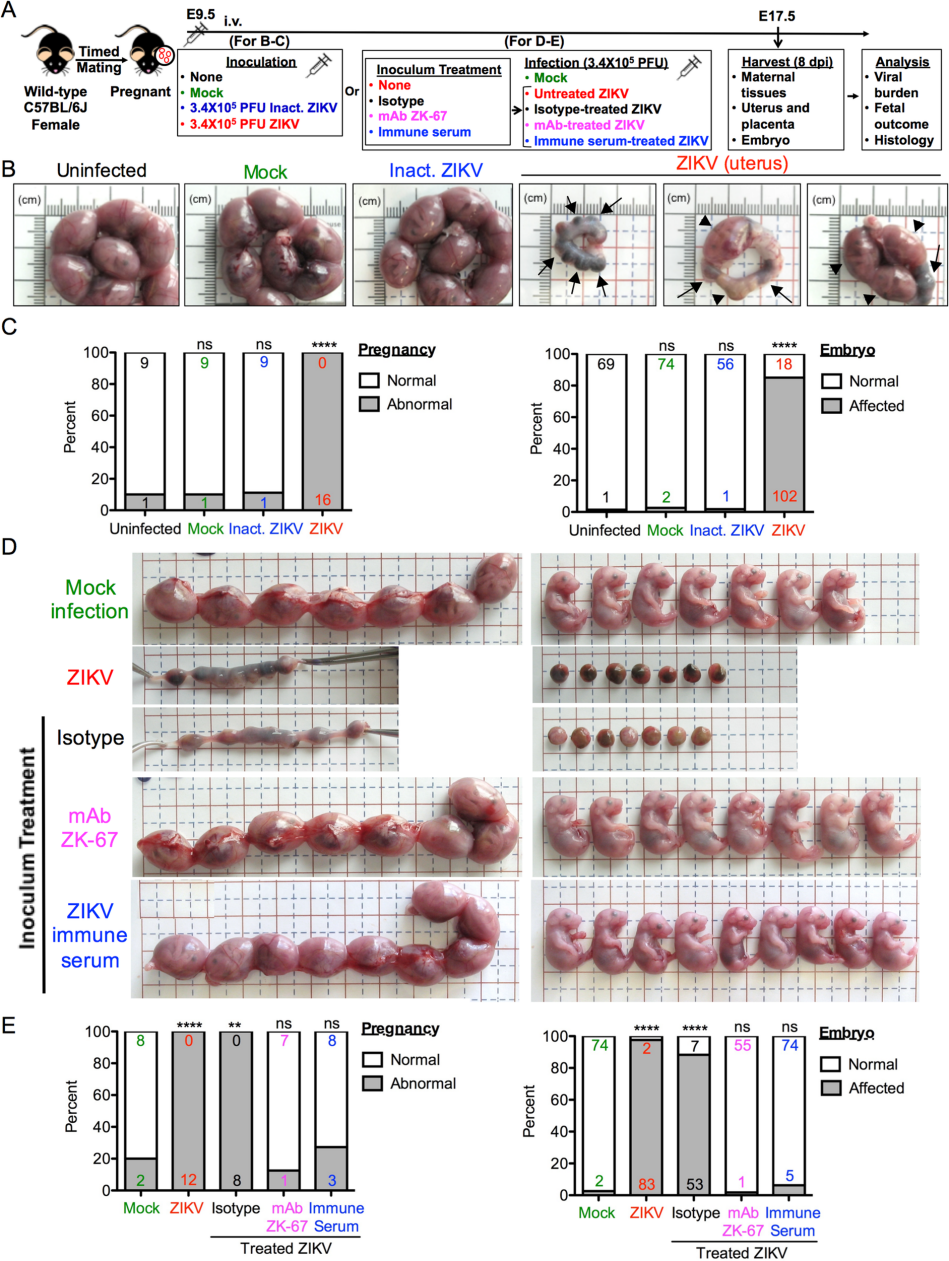
ZIKV infection of pregnant C57BL/6J mice results in fetal demise caused by live infectious virus. (**A**) Scheme of infection and the follow up analyses. For **B** and **C**, pregnant dams at embryonic day 9.5 (E9.5) were infected with 3.4 × 10^5^ PFU of ZIKV by intravenous route. Control mice were left untreated (uninfected), or inoculated with Vero cell culture supernatant (Mock) or 3.4 × 10^5^ PFU-equivalent of heat-inactivated ZIKV (Inact. ZIKV). For **D** and **E**, ZIKV was left untreated (ZIKV) or pretreated with mouse IgG2a isotype (Isotype), the neutralizing anti-ZIKV mAb (mAb ZK-67), or the immune serum harvested from ZIKV-infected adult mice (ZIKV immune serum). Pregnant dams at E9.5 were then infected with 3.4 × 10^5^ PFU-equivalent of treated ZIKV by intravenous route. Control mice were inoculated with Vero cell culture supernatant (Mock). Mice were sacrificed 8 days post infection (dpi) at E17.5, and maternal tissues, placentas and embryos were harvested and examined. (**B**) Representative images of E17.5 uteri. The uteri of ZIKV-infected mice showed complete or partial fetal demise. Arrows indicate the placental residues of resorbed embryos. Arrowheads indicate the remaining intact embryos. (**C**) Impact of ZIKV infection on dams and embryos at E17.5. The percentage of dams showing abnormal pregnancy (i.e. at least one embryo in the litter showed morphological abnormality or suffered demise/resorption) is shown in the left panel. The percentage of embryos that were affected (i.e. had undergone demise/resorption, or exhibited any sign of growth restriction, malformation, or anatomical developmental defect by gross morphological examination) is shown in the right panel. Numbers on bars indicate normal pregnancy/embryo (top) or abnormal pregnancy/affected embryo (bottom). (ns, not significant; **** p<0.0001 compared with numbers in uninfected group by Fisher’s exact test). (**D**) Representative images of uteri and embryos or placental residues carried by dams infected with pretreated ZIKV. The uteri and embryos of dams infected with mAb- or immune serum-treated ZIKV showed no abnormality compared with those of mock infection. (**E**) Impact of infection with pretreated ZIKV on dams and embryos at E17.5, as described in (C). (ns, not significant; ** p<0.01; **** p<0.0001 compared with numbers in Mock group by Fisher’s exact test). Data for all panels are pooled from 5 (B and C) or 2 (D and E) independent experiments.

We infected wild-type, timed-pregnant C57BL/6J mice intravenously with 3.4 × 10^5^ PFU of ZIKV Puerto Rico strain PRVABC59 at embryonic day 9.5 (E9.5) of pregnancy, a time just prior to complete placentation (E10-E10.5). Mice were sacrificed 8 days post infection (dpi) at E17.5, and uteri and individual embryos were evaluated for morphological appearance and weight (scheme outlined in Fig 1A). Mock-infected or mice given heat-inactivated ZIKV showed no apparent differences compared with untreated mice (Fig 1B, left 3 panels). In contrast, the uteri of live ZIKV-infected mice contained embryos undergoing fetal demise (Fig 1B, right 3 panels). In some cases, the embryos had all undergone complete resorption, leaving only the placental residues and embryonic debris inside a constricted amniotic sac. In other cases, dams carried a mixture of placental residues and/or morphologically abnormal embryos. The few embryos that remained exhibited a range of significant growth restriction and malformations, as well as significantly reduced weight (S1 Fig). All 16 ZIKV-infected dams showed abnormal pregnancies in that they all carried at least one or more dead embryos or placental residues, or morphologically abnormal but live embryos (Fig 1C, left panel). Of the 120 implantation sites in the ZIKV-infected dams, 85% were affected, exhibiting complete embryonic resorption (i.e. demise), or signs of growth restriction, malformation, or anatomical developmental defect by gross morphological examination (Fig 1C, right panel). These data demonstrate that this experimental protocol results in a highly reproducible model of profoundly adverse ZIKV-associated fetal outcomes.

To confirm that live infectious ZIKV is the causative agent of these adverse fetal outcomes, we pretreated the inoculum prior to infection with a neutralizing ZIKV-specific mAb, ZK-67 [33], or the immune serum harvested from ZIKV-infected adult mice (scheme outlined in Fig 1A). Results shown in Fig 1D and quantified in Fig 1E show that all of the dams infected with untreated ZIKV or irrelevant isotype-treated ZIKV showed abnormal pregnancies, and more than 90% of their embryos were affected (i.e. demise or abnormal). In contrast, dams infected with ZIKV pretreated with either ZK-67 mAb or immune serum, and their embryos, showed no apparent morphological differences nor statistical significance compared with those inoculated with Vero cell culture supernatant (i.e. mock infection). Together, these data demonstrate that the profound adverse fetal outcomes result from live infectious ZIKV, rather than other immuno-stimulatory molecules that potentially could be present in the inoculum.

### The impact of ZIKV infection on fetal demise depends on initial infection dose and gestational age

**Fig 2:**
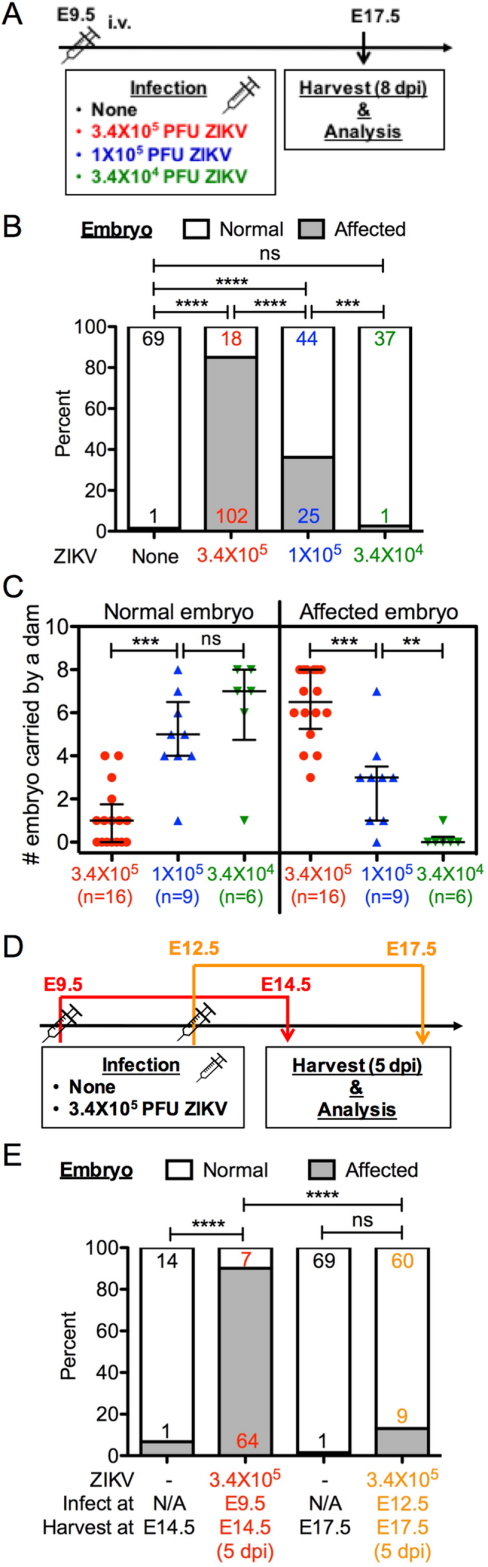
ZIKV-associated adverse fetal outcomes depend on initial dose and gestational age of maternal infection. (**A-C**) Pregnant dams at E9.5 were infected with 3.4, 1, or 0.34 × 10^5^ PFU of ZIKV by intravenous route, and sacrificed 8 dpi at E17.5. Control mice were left uninfected. The uninfected and 3.4 × 10^5^ groups were the same groups as shown in Fig 1C. (**A**) Scheme of infection. (**B**) Impact of ZIKV inoculum dose on embryos, as described in Fig 1C. Numbers on bars indicate normal embryo (top) or affected embryo (bottom). (ns, not significant; *** p<0.001; **** p<0.0001 as compared with numbers between indicated groups by Fisher’s exact test). (**C**) Impact of ZIKV infection on the number of affected embryos carried by each individual dam. Number of dams in each group is listed below dose in the parentheses. Symbols show the number of embryos from each dam with indicated fetal outcomes. Data shown are median with interquartile range. (ns, not significant; ** p<0.01; *** p<0.0001 by Mann-Whitney test). (**D-E**) Pregnant dams were infected with 3.4 × 10^5^ PFU of ZIKV at E9.5 or E12.5, and sacrificed 5 dpi at E14.5 or E17.5, respectively. (**D**) Scheme of infection. (**E**) Impact of ZIKV infection timing on embryos, as described in Fig 1C. (ns, not significant; **** p<0.0001 by Fisher’s exact test). N/A, not applicable.

To determine whether ZIKV infection-induced fetal demise was dose-dependent, and to assess whether disease severity could be modulated by a lower inoculum of virus, we infected pregnant dams at E9.5 with 3.4, 1, or 0.34 × 10^5^ PFU and sacrificed the mice 8 days later (scheme outlined in Fig 2A). As shown in Fig 2B and 2C, we observed a dose-dependent effect of viral inoculum on both the number/percentage of affected embryos (Fig 2B) and the extent to which the embryos carried by individual dams were affected (Fig 2C). While 85% of total embryos carried by the dams infected with 3.4 × 10^5^ PFU were affected, only 36% of total embryos carried by the dams infected with 1 × 10^5^ PFU were affected, which was still significantly higher than that of uninfected dams (Fig 2B). In contrast, less than 3% of total embryos carried by the dams infected with 3.4 × 10^4^ PFU were affected (Fig 2B). As the dose of initial viral inoculation progressively decreased, so did the proportion of morphologically affected embryos to normal embryos carried by each individual dam (Fig 2C). These data indicated a dose-dependent effect of administered viral inoculum on fetal outcome.

To investigate the effect of gestational stage and placental maturity on ZIKV-associated adverse effects, we infected dams with 3.4 × 10^5^ PFU ZIKV at E9.5 or E12.5 and compared maternal and embryonic outcomes at 5 dpi (scheme outlined in Fig 2D). Consistent with our previous observation (Fig 1), we observed adverse fetal outcomes in dams infected at E9.5: 90% of the embryos were affected (Fig 2E). In contrast, when dams were infected at E12.5, only 13% of their embryos were affected (Fig 2E). Thus, the gestational age at which the mice encountered ZIKV dramatically affected the severity of fetal outcome.

### ZIKV viral RNA is detectable in some maternal tissues, placentas and embryos, but its presence is not a reliable indicator of adverse fetal outcomes

**Fig 3:**
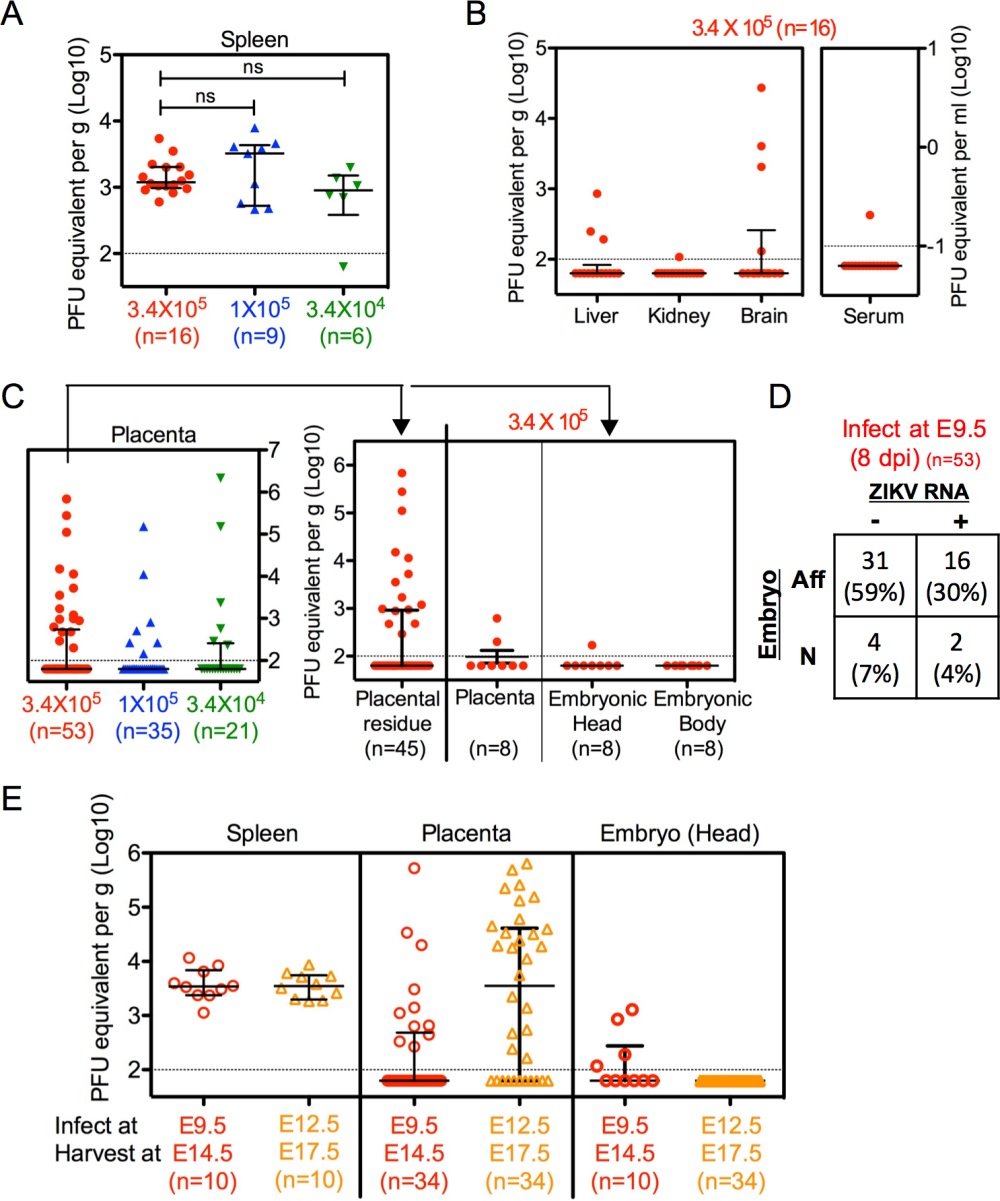
ZIKV RNA is detectible but its presence does not correlate with fetal outcomes. (**A-D**) Pregnant dams at E9.5 were infected with 3.4, 1, or 0.34 × 10^5^ PFU of ZIKV by intravenous route, and sacrificed 8 dpi at E17.5. ZIKV RNA levels were measured by quantitative real-time PCR. (**A**) ZIKV RNA levels in the spleens of dams infected with 3 doses of ZIKV. (**B**) ZIKV RNA levels in the liver, kidney, brain or serum in dams infected with 3.4 × 10^5^ PFU. (**C**) ZIKV RNA levels of all placental samples carried by dams infected with 3 doses (left panel), or placental residues or remaining intact embryos and their corresponding placentas carried by dams infected with 3.4 × 10^5^ PFU (right panel). (**D**) Summary of embryonic outcome in relation to viral RNA presence. The table depicts the numbers and percentages of embryos that were affected (Aff) or normal (N) in the absence (-) or presence (+) of detectable ZIKV RNA by real-time PCR in placentas, corresponding embryos, or both. Affected embryos include those which suffered demise/resorption, or exhibited any sign of growth restriction, malformation, or anatomical developmental defect by gross morphological examination. Chi-square test revealed no statistically significant association between viral RNA presence and embryonic outcome (p = 0.97). (**E**) Pregnant dams were infected with 3.4 × 10^5^ PFU of ZIKV at E9.5 or E12.5, and sacrificed 5 dpi at E14.5 or E17.5, respectively. ZIKV RNA levels in the maternal spleen, placenta or embryonic head were measured by quantitative real-time PCR. (**A-C** and **E**) Data shown are median with interquartile range. Dotted line depicts the limit of detection. (ns, not significant by Mann-Whitney test). Number of samples in each group is listed in the parentheses. Data for all panels are pooled from 2–5 independent experiments.

To investigate the infection status of dams after exposure to ZIKV, we measured ZIKV RNA levels in several maternal tissues (spleen, liver, kidney, brain and serum) by quantitative real-time reverse transcription polymerase chain reaction (RT-PCR). All ZIKV-infected dams exhibited detectable and comparable levels of ZIKV RNA in their spleens, regardless of the initial inoculum dose (Fig 3A). However, ZIKV RNA was infrequently detected in the liver, kidney and brain, and in the serum of a single dam at a very low level when the dams were infected with the highest dose (i.e. 3.4 × 10^5^ PFU) (Fig 3B). None of the dams infected with the two lower doses had detectable levels of ZIKV RNA in the liver, kidney or serum.

To determine the ability of ZIKV to reach the placenta and infect the embryo, we measured ZIKV RNA levels in the placental residues of resorbed embryos, and in the remaining intact embryos and their corresponding placentas. We detected ZIKV RNA in some but not all placentas of dams infected at all three doses (Fig 3C, left panel), indicating the ability of ZIKV to infect the placenta, despite the dams’ intact immunity, and regardless of the ZIKV inoculum dose. In the cases of fetal demise in the dams that were infected with the highest dose, we detected ZIKV RNA in 15 out of 45 placental residues, some of which exhibited high RNA levels (Fig 3C, right panel). However, the remaining 30 of 45 placental residues did not harbor detectable ZIKV RNA, even though all of their corresponding embryos suffered complete resorption (Fig 3C, right panel). Of the 8 remaining embryos that were not reabsorbed, ZIKV RNA was only detectable in a single embryo (head) at a very low level, but not in its corresponding placenta (Fig 3C, right panel). Overall, there was no statistically significant association between presence of detectable ZIKV RNA in the placenta and/or embryo and embryonic morphological outcome (p = 0.97, chi-square test; Fig 3D).

When dams were infected at different gestational age (i.e. E9.5 or E12.5), ZIKV RNA was detectable in all of the maternal spleens after 5 days of infection (Fig 3E). Notably, the levels of ZIKV RNA were comparable regardless of when the dams were infected, implying that the pregnant adult mice are equally susceptible to ZIKV infection at E9.5 and E12.5, even though their embryos show different outcomes (Fig 2E). Interestingly, whereas 32% of the placentas carried by dams infected at E9.5 had detectable ZIKV RNA, 68% of the placentas carried by dams infected at E12.5 had detectable ZIKV RNA (Fig 3E), exhibiting greater placental penetration at E12.5 compared with E9.5. Despite this, no embryos carried by dams infected at E12.5 had detectable ZIKV RNA (Fig 3E). This is consistent with our previous observation (Fig 3D) that ZIKV RNA detected in the dams, placentas or embryos after infection does not associate with adverse fetal outcome; thus, the residual ZIKV RNA level is an unreliable indicator in this model.

### A range of adverse fetal outcome is evident early and corresponds to dosing

**Fig 4:**
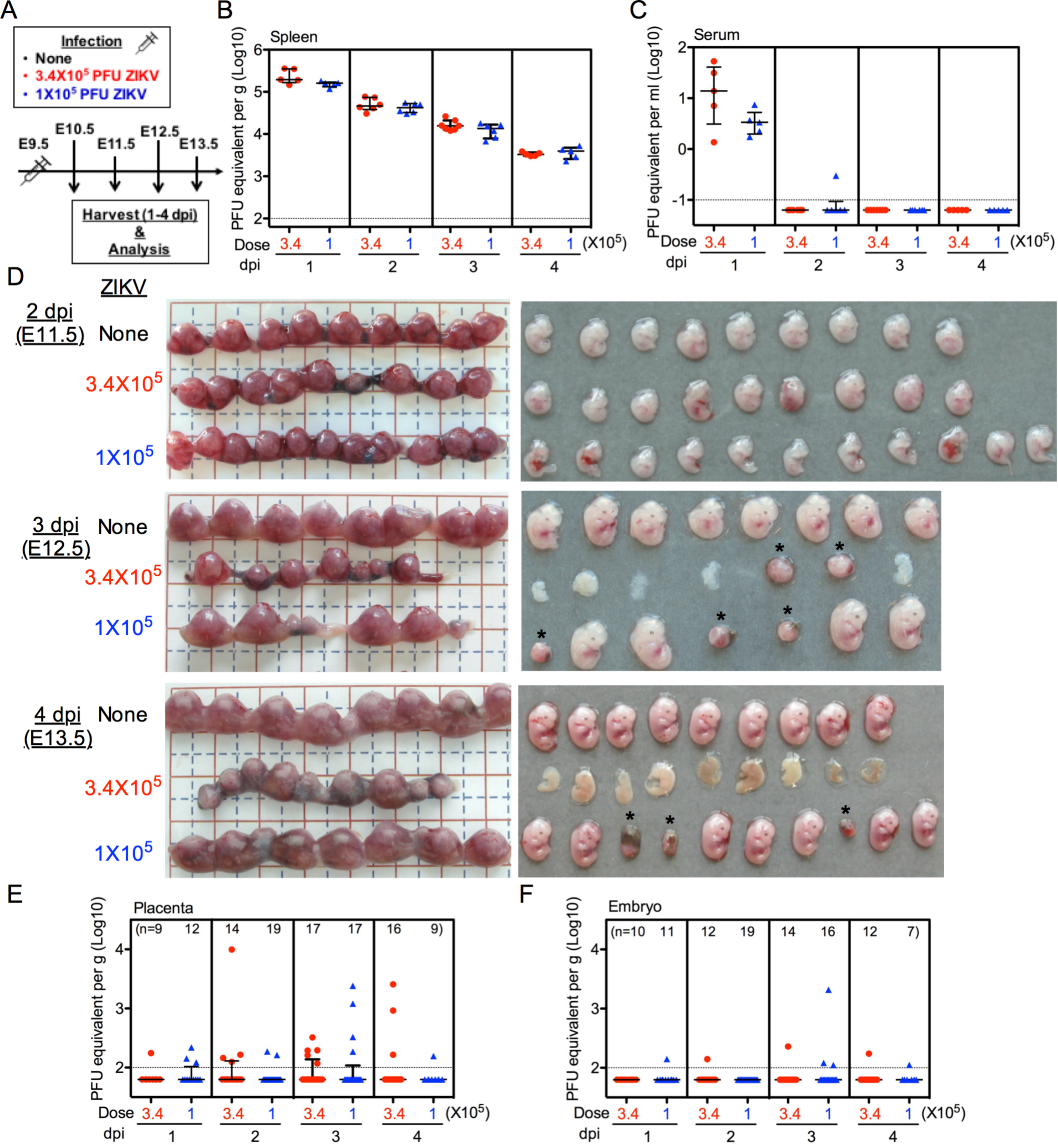
A range of fetal abnormalities present early, and correspond to dosing but not viral RNA level. Pregnant dams at E9.5 were infected with 3.4 or 1 × 10^5^ PFU of ZIKV by intravenous route, and sacrificed at 1 through 4 dpi. Control mice were left uninfected. (**A**) Scheme of infection. (**B** and **C**) ZIKV RNA levels in the spleen (**B**) or serum (**C**) of ZIKV-infected dams. (**D**) Representative images of uteri and embryos at 2–4 dpi. Asterisk indicates the placental residues of demised/resorbed embryos. Fetal abnormalities were observed as early as 2 dpi, with further deterioration over the next 24–48 h to severe fetal demise. (**E** and **F**) ZIKV RNA levels in the placenta (**E**) or whole embryo (**F**). (**B**, **C**, **E**, and **F**) ZIKV RNA levels were measured by quantitative real-time PCR. Dotted line depicts the limit of detection. Data shown are median with interquartile range. Data for all panels are pooled from 2 independent experiments.

The complete fetal demise and resorption observed at 5 or 8 dpi following maternal infection at E9.5 underscores the devastating consequences of maternal ZIKV infection, but does not shed light on the pathophysiologic events that lead to the extreme outcome. The fact that we did not detect ZIKV RNA in all affected placentas and embryos at these time points could not rule out the possibility that fetal morbidity was attributable to ZIKV infection of the placenta and embryo that occurred earlier but was no longer detectible at the time of harvest. To investigate whether early vertical transmission occurs, and whether viral vertical transmission or other early ZIKV-associated events, such as placental pathology, contribute to eventual fetal demise, we infected dams at E9.5 with 3.4 or 1 × 10^5^ PFU ZIKV and sacrificed the mice at 1 through 4 days post infection (scheme outlined in Fig 4A). Consistent with our previous observation at 5 and 8 dpi (Fig 3E and 3A, respectively), all dams harbored ZIKV RNA in the spleens at all earlier time points (Fig 4B). ZIKV RNA levels were the highest in spleens at 1 dpi, then gradually declined. Notably, splenic ZIKV RNA levels did not differ significantly between dams infected with 3.4 or 1 × 10^5^ PFU ZIKV at the various time points. Furthermore, ZIKV RNA was only detected in maternal serum at 1 dpi (Fig 4C), suggesting rapid clearance of ZIKV from the maternal circulation in immunocompetent mice, without establishing prolonged viremia. The levels of virus in the serum in the two infected groups did not differ significantly. Although ZIKV RNA was detected, infectious virus, if present in either maternal spleen or serum, was below the limit of detection of standard plaque assay at all time points, further confirming that the ZIKV infection was limited, yet resulted in adverse fetal outcome.

Gross morphological examination of the uteri and embryos in ZIKV-infected dams revealed visually obvious fetal abnormalities (e.g. growth restriction, growth arrest, and delay of development) as early as 2 dpi, with further deterioration over the next 24–48 h to include severe fetal demise (Fig 4D). At 4 dpi, the embryos carried by dams infected with 3.4 × 10^5^ PFU ZIKV were already frequently discolored and evidently bloodless, and likely non-viable (Fig 4D). Consistent with our previous observation (Fig 2), fetal outcome of dams infected with 1 × 10^5^ PFU ZIKV was less severe than that of dams infected with 3.4 × 10^5^ PFU ZIKV (Fig 4D). Notably, embryonic destruction did not occur synchronously for the entire litter, allowing us to observe varying degrees of early fetal abnormality and overall developmental delay. Furthermore, concordant with our previous observation (Fig 3), ZIKV RNA was detected only sporadically in the placentas (Fig 4E) and embryos (Fig 4F) at these earlier time points. While all embryos were affected at 3 dpi, 70% harbored no detectable ZIKV RNA in the placentas or the embryos, indicating that significant vertical transmission did not occur in the majority of affected embryos, even at earlier time points.

### ZIKV infection results in placental pathology that corresponds to adverse fetal outcome

**Fig 5:**
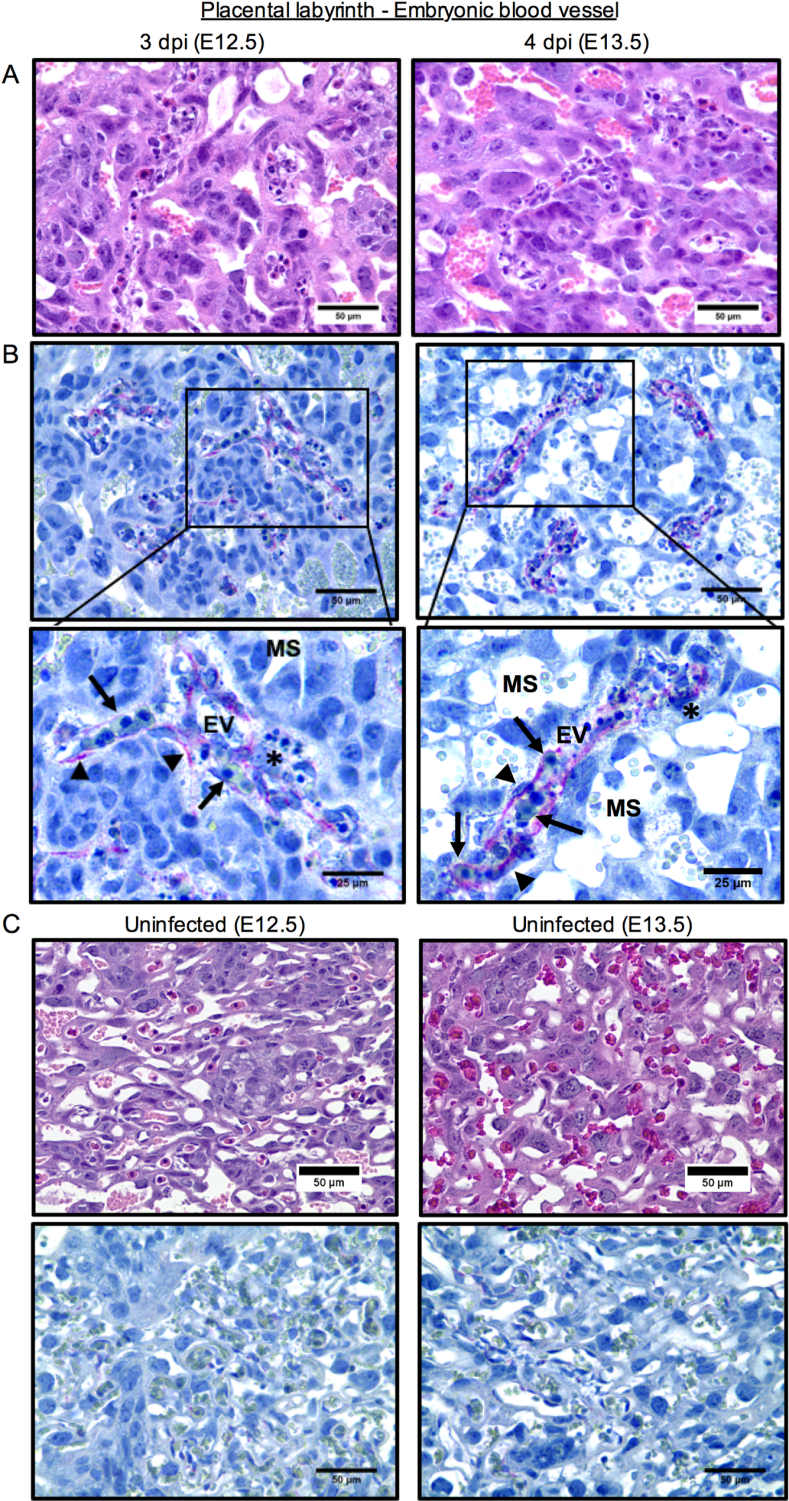
ZIKV antigen co-localizes with embryonic endothelial cells in the placental labyrinth and associates with damage. Pregnant dams at E9.5 were infected with 3.4 × 10^5^ PFU of ZIKV and sacrificed at 3 and 4 dpi. Paraffin-embedded placental tissues were stained with H&E (**A** and **C**) or ZIKV E protein (red) with hematoxylin counterstain (blue) (**B** and **C**). Representative images of the embryonic blood vessels in the placental labyrinth are shown. (**A**) Necrosis within the embryonic blood vessels. (**B**) ZIKV antigen was detected within the embryonic endothelial cells in embryonic blood vessels of the placental labyrinth. Arrows indicate nucleated embryonic red blood cells. Arrowheads indicate the staining of ZIKV antigen and highlight morphology of infected cells and antigen staining pattern. Asterisks indicate areas of necrotic cell debris. (**C**) Tissues from uninfected dams at the same gestational age were used as negative controls. MS, maternal sinus; EV, embryonic blood vessel. Scale bar, 50 μm or 25 μm.

Our analysis thus far revealed that ZIKV RNA level in the placenta or embryo did not predict adverse fetal outcome, even at earlier time points. Subsequently, we looked for histopathological lesions in the placenta that may explain fetal demise at 3 and 4 dpi. We found necrosis of embryonic endothelium within the embryonic blood vessels in the placental labyrinth (Fig 5A). ZIKV envelop (E) protein antigen was detected within the endothelium of embryonic blood vessels in the placental labyrinth but not in embryonic cells or tissues at any time studied (Fig 5B). The linear “flat” antigen staining pattern strongly suggested that ZIKV antigen localized predominantly in the long and thin embryonic endothelial cells, which line the embryonic blood vessels and have flat oval-shaped nuclei. In addition, necrotic cell debris within the embryonic vasculature often accompanied viral staining (Fig 5B), suggesting that placental and embryonic blood circulation may have been compromised, most likely due to ZIKV exposure and subsequent destruction of embryonic endothelial cells. This cellular debris may have originated from necrotic endothelium and other cells.

**Fig 6:**
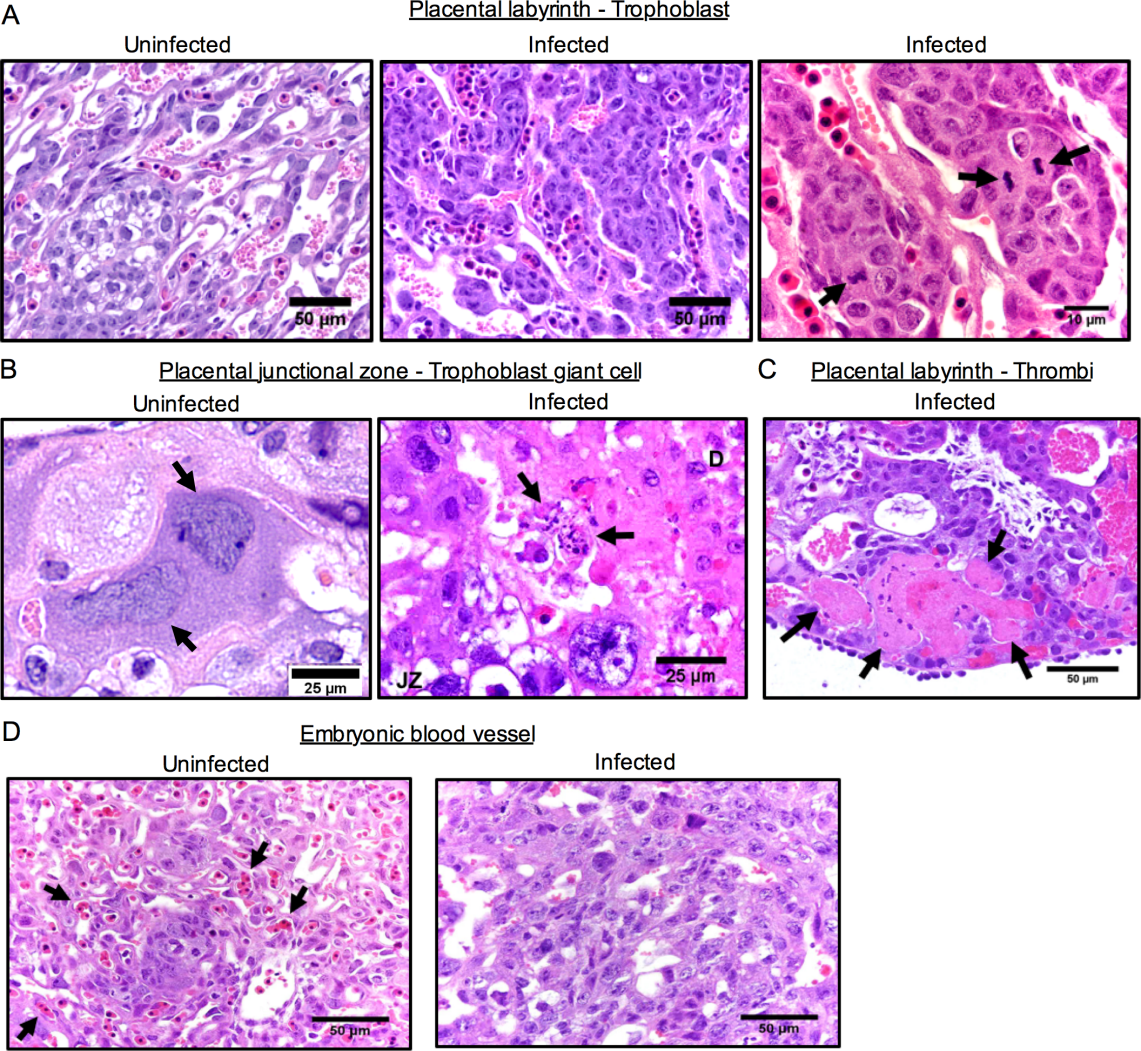
Maternal ZIKV infection induces placental damage. Pregnant dams at E9.5 were infected with 3.4 × 10^5^ PFU of ZIKV and sacrificed at 3 dpi. Paraffin-embedded placental tissues were stained with H&E. Representative images are shown. (**A**) Labyrinth trophoblast hyperplasia in the placentas of infected dams. Arrows indicate mitotic labyrinth trophoblasts. Scale bar, 50 μm or 10 μm. (**B**) Trophoblast giant cell necrosis in the junctional zone. Arrows indicate normal tissue (left) or necrotic cell debris (right). D, decidua; JZ, junctional zone. Scale bar, 25 μm. (**C**) Thrombi in maternal or embryonic blood vessels in the labyrinth. Arrows indicate thrombi. Scale bar, 50 μm. (**D**) Loss of embryonic blood vessels, characterized by the absence of nucleated embryonic red blood cells, in the labyrinth of infected dams. Arrows indicate nucleated embryonic red blood cells. Scale bar, 50 μm.

General histological examination of the placentas associated with morphologically abnormal embryos, regardless of whether viral antigen was detected or not, revealed that the most prominent placental pathological findings were focal or diffuse labyrinth trophoblast hyperplasia (Fig 6A), and trophoblast giant cell degeneration and necrosis in the junctional zone (Fig 6B). We also observed localized areas of necrotic cells and thrombi in the maternal blood spaces in the labyrinth of at least one placenta (Fig 6C). Trophoblast hyperplasia within the labyrinth was evident, with focal or diffuse labyrinth trophoblast basophilia and mitotic trophoblasts (Fig 6A) in close proximity to embryonic blood vessels and maternal blood spaces (trophoblast-lined sinusoids). Necrotic cell debris indicated focal necrosis within the junctional zone (Fig 6B). We consistently observed these features as early as 1 dpi, implying that they may not have been caused by direct viral infection of placental cells. Moreover, while focal necrosis in the labyrinth was seen at 2 dpi, embryos began to show some focal necrosis only at 3 and 4 dpi, indicating that the placental lesions developed prior to those in the embryos. Although the placental necrosis may not have been severe enough to alone cause placental failure, the major anatomical lesions, labyrinth trophoblast hyperplasia and loss of embryonic blood vessels (Fig 6D) after endothelial necrosis likely interfered with embryonic nutrition exchange sufficiently to cause hypoxia to the embryo. Taken together, these data suggest that following maternal ZIKV exposure the normal placental architecture and function was substantially disrupted, causing placental insufficiency leading to fetal demise, even without widespread direct viral infection of the embryos.

**Fig 7:**
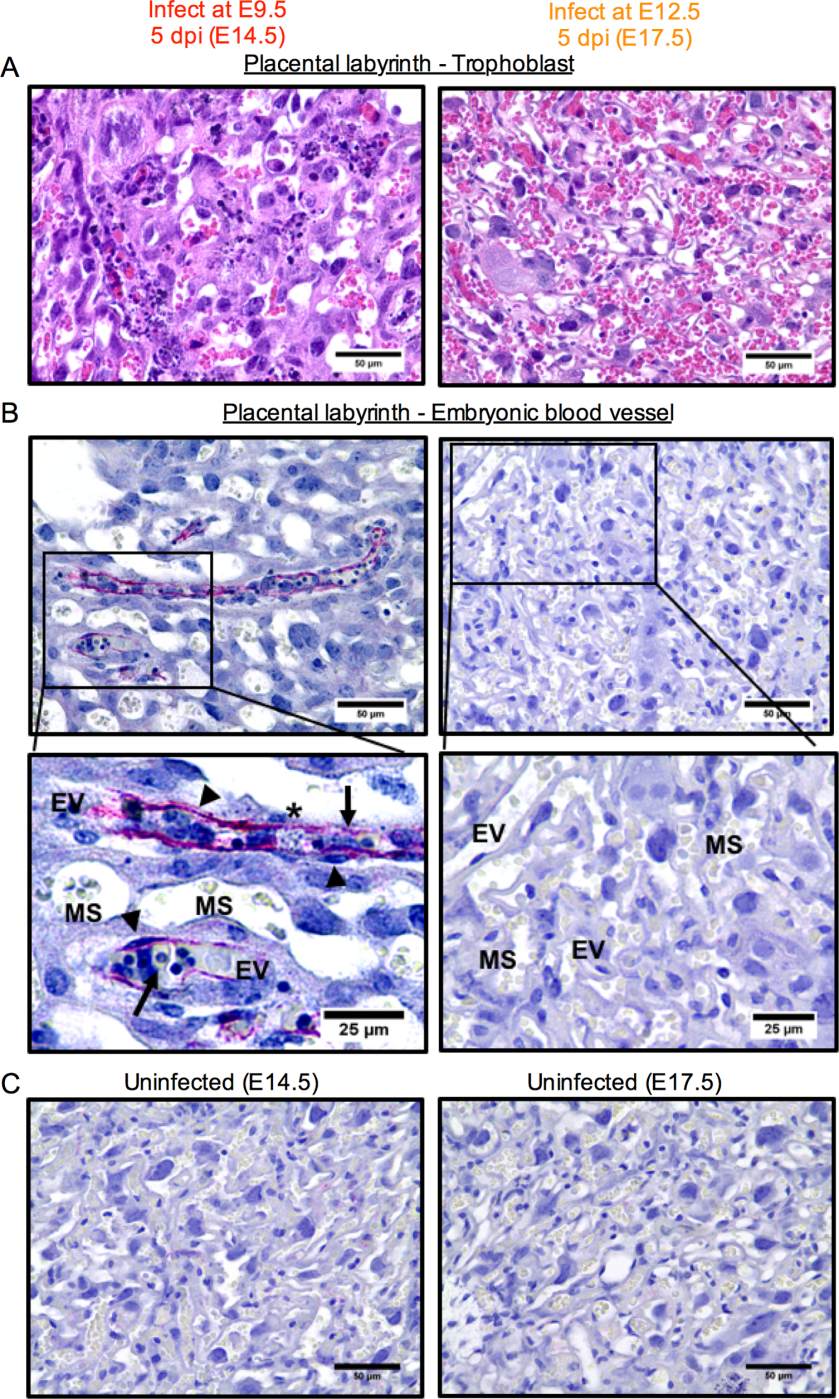
Pathology of the placentas of ZIKV-infected dams depends on the time of exposure during gestation. Pregnant dams were infected with 3.4 × 10^5^ PFU of ZIKV at E9.5 or E12.5, and sacrificed 5 dpi at E14.5 (left panels) or E17.5 (right panels), respectively, as illustrated in Fig 2D. Representative images of placental labyrinth stained with H&E (**A**) or ZIKV E protein (red) with hematoxylin counterstain (blue) (**B** and **C**) are shown. Necrosis and viral antigen were observed in the labyrinth of dams infected at E9.5, but not in dams infected at E12.5. Arrows indicate nucleated embryonic red blood cells. Arrowheads indicate the ZIKV antigen stain and highlight morphology of infected cells and antigen staining pattern. Asterisks indicate areas of necrotic cell debris. (**C**) Tissues from uninfected dams at the same gestational age were used as negative controls for viral staining. MS, maternal sinus; EV, embryonic blood vessel. Scale bar, 50 μm or 25 μm.

In further support of this view, histological evaluation consistently indicated there were no significant differences in the placentas between uninfected dams and dams infected at E12.5 (Fig 7). This is consistent with their normal embryonic morphology we observed at 5 dpi in these dams (Fig 2E). There were no signs of trophoblast hyperplasia or necrotic/apoptotic cells in the labyrinth of the placentas from the dams infected at E12.5 (Fig 7A and 7B, right panels), compared with those from the dams that were infected at E9.5, which showed substantial lesions (Fig 7A and 7B, left panels). These data indicate that placentas and embryos at E9.5 may be more vulnerable and thus exhibit greater pathology than those at E12.5, which appear relatively unaffected. While real-time PCR detected ZIKV RNA in the placental tissue infected at E12.5 (Fig 3E), we were unable to detect ZIKV antigen by immunohistochemistry (Fig 7B).

Placentas in dams infected at E12.5 appeared relatively healthy (Fig 7) despite their higher ZIKV RNA levels (Fig 3E). This, combined with the observation that neither placental nor embryonic ZIKV RNA levels were reliable predictors of fetal outcome (Figs 3 and 4), suggests that placental health is a more meaningful prognosticator of adverse fetal outcome than level of placental or embryonic ZIKV RNA. However, it should be stressed that the placental pathology observed in our model has yet to be directly linked with placental insufficiency as a cause of the high frequency of fetal demise.

### Convalescent immune serum transfer demonstrates that fetal morphological outcome is a useful readout for assessing efficacy of potential protective measures

**Fig 8:**
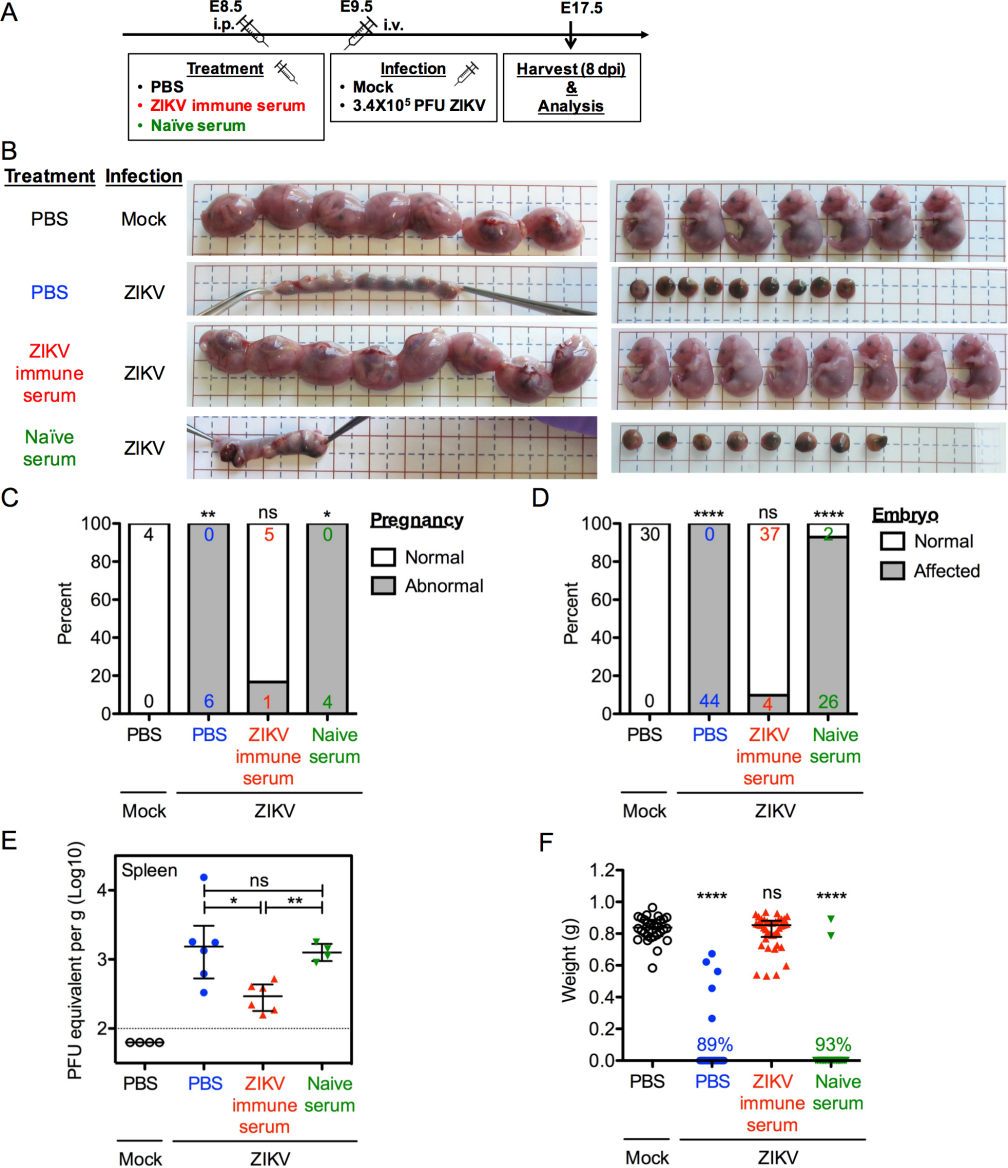
Convalescent immune serum transfer demonstrates the utility of fetal morphological outcome in assessing protective efficacy. Pregnant dams at E8.5 were treated intraperitoneally with PBS, 50 μL of serum from ZIKV-infected mice (ZIKV immune serum), or 250 μL of serum from naïve mice (naïve serum). Dams were infected at E9.5 with 3.4 × 10^5^ PFU of ZIKV by intravenous route, and sacrificed 8 dpi at E17.5. Control mice were injected with Vero cell culture supernatant (Mock). (**A**) Scheme of infection. (**B**) Representative images of uteri and embryos or placental residues. (**C** and **D**) Impact of serum treatment on dams (**C**) or embryos (**D**), as described in Fig 1C. Numbers on bars indicate normal pregnancy/embryo (top) or abnormal pregnancy/affected embryo (bottom). (ns, not significant; * p<0.05; ** p<0.01; **** p<0.0001 compared with numbers in Mock group by Fisher’s exact test). (**E**) ZIKV RNA levels in the spleen of ZIKV-infected dams as measured by quantitative real-time PCR. Data shown are median with interquartile range. Dotted line depicts the limit of detection. (ns, not significant; * p<0.05; ** p<0.01 by Mann-Whitney test). (**F**) Embryonic weight. The percentage indicates the embryos that had undergone complete resorption. Data shown are median with interquartile range. (ns, not significant; **** p<0.0001 compared with Mock group by Mann-Whitney test). Data for all panels are one representative of 2 independent experiments with 4–6 pregnant dams in each group in each experiment.

To demonstrate the utility of our model and to test whether the fetal morphological outcome could serve as a rapid readout for initial screening of candidate vaccines and therapeutics against ZIKV infection, we performed proof-of-principle studies using a potential protective therapy—the immune serum harvested from convalescent ZIKV-infected adult mice. When pregnant dams received immune serum prior to infection (scheme showed in Fig 8A), their uteri and embryos showed no apparent morphological difference compared with mock infected dams, while dams that received saline or naïve serum showed complete fetal demise (Fig 8B). Immune serum significantly decreased the number and percentage of affected dams (Fig 8C) and embryos (Fig 8D) to the level that was comparable to the mock infection. It also significantly reduced ZIKV RNA in the maternal spleens at 8 dpi (Fig 8E). Moreover, the weights of embryos carried by the dams that received immune serum did not differ significantly from those carried by the dams that received mock infection (Fig 8F). Together, along with results shown previously in Fig 1D and 1E, these results indicate that immune serum could prevent adverse fetal outcome in the pregnancy setting, and demonstrate that in this model, embryonic viability and gross uterine and embryonic morphology can serve as rapid and easy *in vivo* phenotypic readouts for the early screening of the efficacy of candidate vaccines and therapeutics against ZIKV infection.

### Early postnatal deaths among newborns carried by dams infected with ZIKV at E12.5

**Fig 9:**
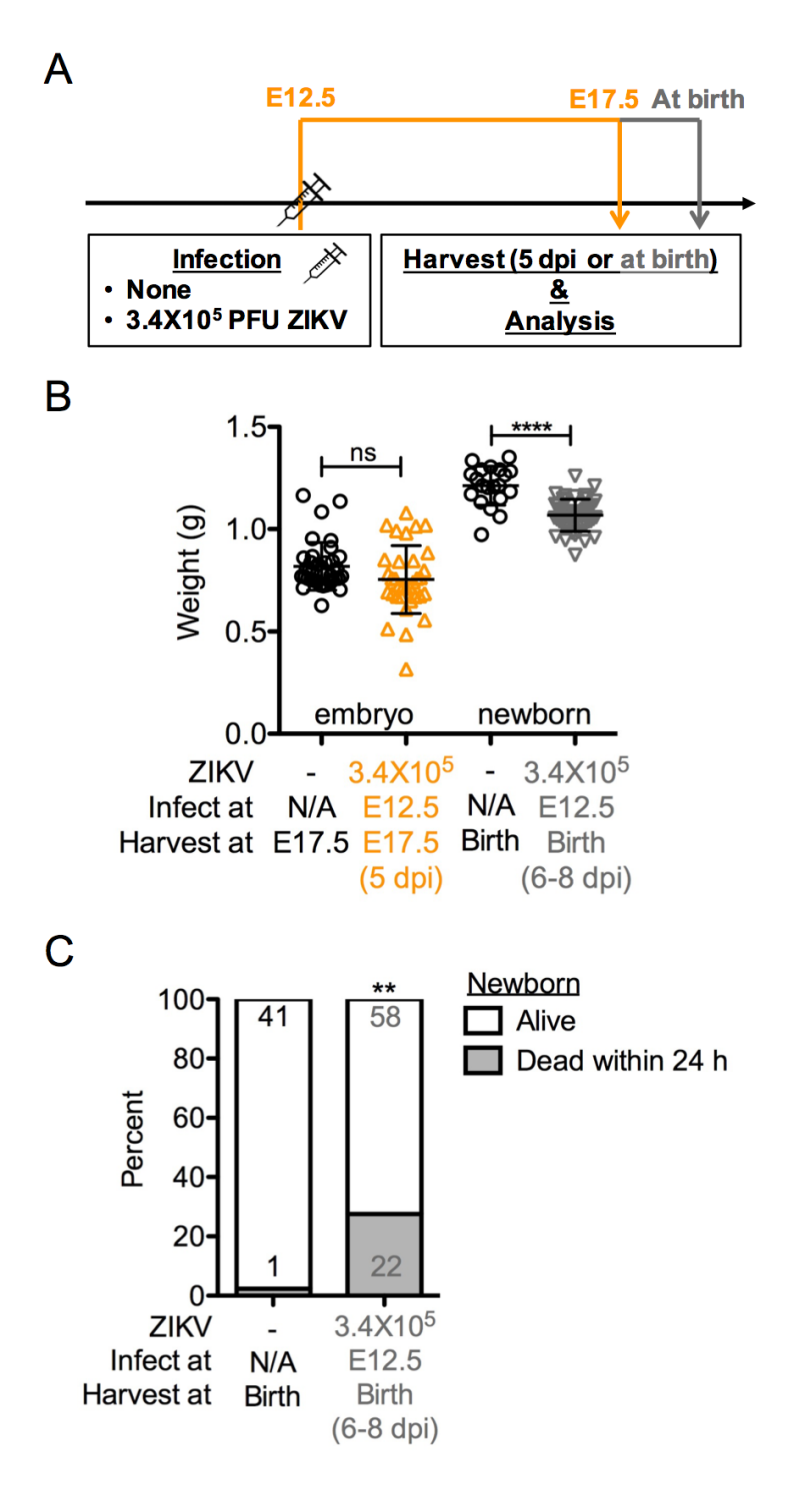
Newborns of dams infected at E12.5 exhibit a significant rate of early postnatal death. Pregnant dams were infected with 3.4 × 10^5^ PFU at E12.5, and sacrificed 5 dpi at E17.5 or allowed to carry to term. Both dams and newborns were examined within 24 h after giving birth. (**A**) Scheme of infection. (**B**) E17.5 embryo and newborn weight. Data shown are mean with SD. (ns, not significant; **** p<0.0001 compared with the uninfected group by Student *t* test) (**C**) Newborn viability. Numbers on bars indicate newborns that were alive (top) or born alive but dead within 24 h after birth (bottom). (ns, not significant; ** p<0.01 compared with numbers in uninfected group by Fisher’s exact test). Data for all panels are pooled from 2–3 independent experiments. N/A, not applicable.

It is important to mention that despite observing no overt embryonic abnormalities 5 days after an E12.5 maternal infection (Fig 2E), we cannot conclude that those embryos will be spared from future pathological consequences. Indeed, when a parallel cohort of dams was allowed to carry their pregnancies to term (scheme showed in Fig 9A), we observed significant reductions in weights of newborns carried by ZIKV-infected dams (Fig 9B), as well as markedly increased neonatal morbidity within 24 h of birth (Fig 9C). We do not yet know whether this was a postnatal effect on the newborns from ZIKV exposure *in utero* or just maternal negligence and/or destruction of newborns that were undersized, perhaps resulting from maternal virus exposure. Nevertheless, our findings suggest that in this immunocompetent mouse model, maternal exposure to ZIKV at later stages of pregnancy may still pose risks of adverse postnatal sequela, even in the absence of acute fetal pathology *in utero* or at birth.

## Discussion

We report a highly reproducible pregnancy model in ZIKV-infected immunocompetent C57BL/6J mice, exhibiting a high incidence of adverse pregnancy outcomes including profound fetal morphological abnormalities and fetal demise, in the absence of viral vertical transmission. This model revealed fetal pathology, placental pathology, and potential postnatal impact. There are several key observations from this study. First, ZIKV infection of pregnant immunologically intact mice at E9.5 caused dramatic deleterious fetal outcomes. This was caused by infectious virus and depended on initial viral dose administered. Second, placental pathology was consistently observed in affected concepti, independent of detectable ZIKV RNA or protein in the placentas and/or embryos. Third, whereas pregnant immunocompetent females themselves resisted effects of ZIKV infection, their embryos appeared exquisitely sensitive to infection, and this effect depended on the time of infection during gestation. Fourth, even in the absence of acute fetal pathology *in utero* or at birth, pups born from ZIKV-infected dams may nonetheless be adversely affected.

Our findings argue against the concept that ZIKV-associated adverse fetal outcome is simply the result of virus crossing the placenta and having direct effects on the fetus. Rather, we show that adverse fetal outcome can occur in the apparent absence of significant, direct fetal infection. Our data support a model of placental insufficiency as a contributing cause of fetal demise in an immunocompetent setting. Moreover, Yockey et al. showed that placental damage with the potential to harm the embryo can result from Type I IFN-triggered immunopathology [34]. Taken together, these findings are in agreement with studies in pregnant rhesus macaques and humans, which reported that the severity of fetal outcome did not generally correlate with clinical maternal disease severity, the ZIKV RNA level in the serum or urine, or the duration of maternal viremia [28, 35], and raise the possibility that maternal disease outcome may not be strictly predictive of fetal outcome.

Humans usually contract ZIKV during mosquito feeding, which has been estimated to deliver between 10^4^ to 10^6^ PFU in related flaviviruses WNV and DENV [36, 37]. Although the doses we administered were within this range, the possibility exists that the responses induced by experimental intravenous infection may differ from those induced by mosquito bites in humans. Indeed, a recent publication found that infection via mosquito bite delays ZIKV replication as compared to needle inoculum and alters ZIKV tissue tropism in rhesus macaques [38]. In addition, flaviviruses are known to antagonize Type I IFN signaling by targeting the key component STAT2 in order to replicate and cause human disease [24]. Unlike humans, mice are resistant to this mechanism, hence ZIKV is unable to replicate efficiently in immunocompetent mice. Our observations that ZIKV was rapidly cleared from maternal circulation and gradually declined in maternal spleen suggest a non-productive infection where after initially infecting host cells ZIKV may not replicate, or may replicate but fail to subsequently release infectious virus. Failure to establish prolonged viremia and cause vertical transmission during pregnancy differs from the common outcome of humans and nonhuman primates. However, our data suggest that the impact of ZIKV on the embryo can result from even a non-productive infection, highlighting the extreme sensitivity of the pregnancy process.

Significantly, even in the case of detectable ZIKV RNA in maternal and fetal tissues, replicating infectious ZIKV are not always detectable in maternal or fetal tissues by standard plaque assay, as reported in a pregnant nonhuman primate study where fetal brain damage was observed [12]. Using a two-step enhanced plaque assay where virus is pre-amplified in insect cells prior to performing a standard plaque assay, we have successfully detected infectious viruses in the placentas of dams infected at E9.5 with 3.4 × 10^5^ PFU ZIKV at 5 and 8 dpi (13% and 8%, respectively). Although the detection frequency was low, it implies that sporadic or modest productive infection is still possible even in these immunocompetent pregnant mice. Notably, our enhanced plaque assay failed to detect any infectious virus in maternal spleen at 5 and 8 dpi, suggesting that replication-competent infectious viruses may take sanctuary in the placenta, even when they are unable to persist elsewhere in immunocompetent mice. The abovementioned limitations may affect the direct application of our findings to human infection. However, our findings nonetheless demonstrate the potential for even non-productive ZIKV infection to cause critical fetal outcomes during pregnancy, and provide a useful model to investigate the underlying mechanisms.

Despite inherent limitations, our data illustrate the importance of an immunocompetent mouse pregnancy model. In prior animal models where vertical transmission was demonstrated and believed to be required for adverse fetal outcome [6, 7, 19], the use of Type I IFN blockade or the localized inoculation directly into the vaginal tract or uterus induced robust maternal viremia and/or direct viral seeding of reproductive tissues proximal to the conceptus. The resulting profound viral replication in the placenta was a likely driver of universal corresponding embryonic infection. The rampant viral infection in those studies presumably obscured the opportunity to identify other determinants of embryonic distress. In contrast, our use of systemically infected immunocompetent mice allowed the establishment of a sub-maximal level of infection that is subtle enough to observe previously unnoticed nuances of placental pathology. Our observation that adverse fetal outcome may occur even in the absence of vertical transmission suggests that ZIKV countermeasures that can effectively block vertical transmission still may not guarantee prevention of adverse fetal outcome.

The placenta forms a crucial physical barrier between the maternal and fetal compartments, preventing pathogen transmission during pregnancy. It also mediates exchange of gases, nutrients and wastes. Previous mouse models have demonstrated that ZIKV was detected in the placenta and resulted in trophoblast infection and apoptosis, vascular damage, hemorrhage, and loss of placental structure [6, 7, 19]. This pathology could compromise placental barrier function to allow transplacental transmission, as well as disrupt exchange function, leading to placental insufficiency. In cases where we observed virus in the placenta, we also observed that ZIKV antigen was co-localized within normal and necrotic labyrinth embryonic endothelial cells, supporting the possibility that ZIKV infection directly damages the placenta and reduces its circulation function. However, the majority of the placental and fetal pathology observed in our model showed infrequent or no measurable associated viral infection, suggesting that placental function may be compromised despite negligible viral titer. Indeed, a pregnancy mouse model of murine γ-herpes virus 68 (MHV-68) infection suggested that even in the absence of vertical transmission, the fetus could be adversely affected by an inflammatory response induced by viral invasion of the placenta [39].

In immunocompetent mice, it is conceivable that the maternal (or even fetal) immune response could cause collateral damage to the placenta in the course of viral clearance, resulting in immunopathology and placental insufficiency without detectable virus. Subsequently, such damage could in turn facilitate viral breach of the placental barrier in cases where the maternal infection has not been resolved. ZIKV infection has been shown to provoke an antiviral immune response in both human Hofbauer cells and mouse placenta [19, 40]. Much remains to be learned about the possible protective and detrimental roles of maternal and fetal innate and adaptive immunity against ZIKV during pregnancy. A recent publication suggested that fetal Type I IFN signaling may play a detrimental role in mediating fetal demise after ZIKV infection by causing abnormal placental development [34]. Using a sophisticated breeding scheme and vaginal infection, it showed that only the embryos with functional Type I IFN signaling were resorbed despite relatively lower viral titer in their placentas, while their littermates with defective Type I IFN signaling continued to develop. It further showed that concepti with functional Type I IFN signaling had increased apoptotic cells in the placental labyrinth and upregulated hypoxia response genes in the embryo. Consistent with our observations, it supports the notion that placental dysfunction resulting from innate immune response is a possible cause of fetal demise, and taken together may provide insight into the underlying mechanisms of adverse fetal outcome of ZIKV infection in immunocompetent mice. However, whether the Type I IFN signaling-mediated placental pathology in immunocompetent hosts is specific to ZIKV infection or a general collateral damage after viral infection warrants further investigation.

The most consistent pathological feature associated with fetal abnormality in our study was trophoblast hyperplasia and embryonic endothelial cell necrosis in the labyrinth leading to loss of labyrinth embryonic blood vessels, and focal necrosis in the junctional zone. Trophoblast hyperplasia in the labyrinth has been shown in an infection-unrelated study to lead to global disruption of labyrinth and vascular architecture, and ultimately fetal death [41]. Our data suggest that trophoblast hyperplasia is not directly caused by viral infection, as placentas from dams infected at E9.5 exhibited trophoblast hyperplasia, while those from dams infected at E12.5 did not, despite high placental ZIKV RNA levels. Although the direct link between trophoblast hyperplasia and fetal abnormality/demise remains to be established, our study clearly indicates that placental pathology can contribute to ZIKV-associated adverse fetal outcome independent of direct viral presence within developing embryos in immunocompetent mice.

The devastating effects of ZIKV infection during pregnancy on fetal development makes it clear that a pregnancy model is necessary for pre-clinical testing of vaccines and therapeutics. Protective efficacy of experimental vaccines against ZIKV-induced fetal demise was reported in a mouse pregnancy model using Type I IFN blockade [42]. Our immunocompetent pregnancy model could provide an additional methodologically simple and high throughput platform for pre-clinical testing. However, as we have demonstrated that the absence of detectable virus does not necessarily predict fetal health, and birth of pups does not guarantee their survival, longer term postnatal follow-up monitoring in both models is necessary to confirm the health or survival of newborns. Moreover, it has been shown that ZIKV can persist at low levels in several anatomically compartmentalized areas, including the central nervous system, reproductive tracts or bodily fluids for up to several weeks [43–45], which may be difficult to detect with standard RT-PCR or plaque assays. Similarly, placental tissue may provide a refuge where ZIKV may avoid complete clearance and persist at trace levels. Indeed, our ability to detect replication-competent virus at 5 dpi in placenta using an enhanced plaque assay, despite its absence in spleens, implies that the placenta may be a uniquely susceptible sanctuary for ZIKV compared with other tissues. Moreover, the profound harmful effects of infection or immunity on the fetus while leaving the mother essentially spared reveals the exquisite sensitivity of the placental-fetal environment to infection. Therefore, it is critical to determine not only whether a vaccine or drug successfully protects against overt disease/infection in adults, but also prevents fetal sequelae during pregnancy.

## Materials and methods

### Ethics statement

All animal studies were conducted in accordance with the Guide for Care and Use of Laboratory Animals of the National Institutes of Health and approved by Trudeau Institute Animal Care and Use Committee (IACUC protocol # 16–009)

### Viruses, cells and titration

The ZIKV Puerto Rico strain PRVABC59 was obtained through BEI Resources, NIAID, NIH (NR-50240). Vero cells (African green monkey kidney epithelial cells) were purchased from American Type Culture Collection (ATCC CCL-81) and maintained in DMEM supplemented with 10% heat-inactivated FBS, 2mM L-glutamine, 1mM penicillin-streptomycin and 0.3% sodium bicarbonate at 37°C with 5% CO_2_.

ZIKV stocks were propagated in low passage number of Vero cells infected at a multiplicity of infection (MOI) of 0.1. Culture supernatants from both mock-infected and ZIKV-infected cells were harvested 4 days after infection and clarified by centrifugation at 3800*g* for 15 min at 4°C. Fetal bovine serum was added to 20% final concentration (v/v) and aliquots were stored at -80°C. The infectious viral titer of stocks was determined by plaque assay on Vero cell monolayers. Briefly, Vero cells were seeded in 6 well plate to reach confluency in 18–24 h. Cells were infected with 1 ml serial 10-fold dilutions of supernatant prepared in neat DMEM for 1 h at 37°C. Then cells were overlaid with carboxymethyl cellulose medium (0.75% in Vero cell medium) and incubated for 5 days at 37°C. Cells were fixed with methanol and stained with crystal violet to visualize the plaques. The most appropriate dilution was chosen to determine the amount of infectious virus in the stocks.

### Mice and infections

Female C57BL/6J mice were purchased from Jackson Laboratory (Bar Harbor, ME). Mice were set up for timed-mating at 8–12 weeks of age and the day the plug was found was considered as 0.5 day of gestation (i.e. E0.5).

At indicated gestational days, pregnant mice were lightly anesthetized with isoflurane and infected with 3.4 × 10^4^, 1 × 10^5^, or 3.4 × 10^5^ PFU of ZIKV in a volume of 100 μL by intravenous (retro-orbital) route. Control mice were left untreated, or inoculated with 100 μL of Vero cell culture supernatant (mock infection) or 3.4 × 10^5^ PFU-equivalent of heat-inactivated or pretreated ZIKV. Some pregnant mice were allowed to give birth, and both the dams and newborns were examined within 24 h after birth.

To prepare heat-inactivated ZIKV, viral stock was heated to 60°C for 1 h. To pretreat inoculum prior to infection, viral stock was incubated with 0.5 mg/mL of the neutralizing anti-ZIKV mAb ZK-67 [33] (Absolute Antibody Ltd, Oxford, UK), 0.5 mg/mL of mouse IgG2a isotype control (clone C1.18.4; BioXCell, West Lebanon, NH) or 1:4 of ZIKV-immune serum per 3.4 × 10^5^ PFU at 37°C for 1 h. An 3.4 × 10^5^ PFU-equivalent of pretreated ZIKV was administered intravenously to pregnant mice. The viability and infectivity of ZIKV after treatment was confirmed by plaque assay.

To generate convalescent-phase serum for passive transfer, female C57BL/6J mice at >10 weeks of age were infected intravenously with 2 × 10^5^ PFU of ZIKV at day 0 and day 35, and serum samples were collected at day 50, pooled, aliquoted and stored at -80°C. Control serum was collected from uninfected naïve mice at the same age. Pregnant mice at E8.5 were treated intraperitoneally with 50 μL of immune serum, 250 μL of naïve serum or saline in a total volume of 250 μL. Mice were then infected intravenously with 3.4 × 10^5^ PFU of ZIKV. Control mice were inoculated with 100 μL of Vero cell culture supernatant (mock infection).

### Measurement of viral burden

ZIKV-infected pregnant mice were euthanized at indicated times after infection. Spleen, liver, brain, kidney, as well as embryos and their corresponding placentas were harvested, snap-frozen in liquid nitrogen, or homogenized with stainless steel beads in 1 ml of DMEM supplemented with 2% heat-inactivated FBS using a TissueLyzer II instrument (QIAGEN). All samples were stored at -80°C until RNA purification or virus titration. Some placental and embryonic samples were fixed in 10% neutral buffered formalin for further histological analysis.

To measure infectious viral particles, tissue homogenates were thawed and clarified by centrifugation at 2000*g* for 10 min at 4°C. Viral burden was determined by plaque assay on Vero cells as described above. To measure ZIKV RNA levels, frozen tissue samples were disrupted and homogenized in Buffer RLT (QIAGEN) containing β-Mercaptoethanol (β-ME) using a Tissuemiser homogenizer (Fisher Scientific) and total RNA was purified using RNeasy Mini Kit (QIAGEN) according to the manufacturer’s instructions. Total RNA was reverse-transcribed to cDNA using random hexamers and ZIKV RNA levels were determined by quantitative real-time PCR on a 7500 Fast Real-time PCR System (Applied Biosystems) using standard cycling conditions. The ZIKV-specific primer set and probe were previously published [46]: forward, 5’-CCGCTGCCCAACACAAG-3’, reverse 5’-CCACTAACGTTCTTTTGCAGACAT-3’, probe 5’-/56-FAM/AGCCTACCT/ZEN/TGACAAGCAGTCAGACACTCAA/3IABkFQ/-3’ (Integrated DNA Technologies). Levels of PCR product were normalized to the housekeeping gene GAPDH: forward 5’-CTCGTCCCGTAGACAAAATGG-3’, reverse 5’-AATCTCCACTTTGCCACTGCA-3’, and probe CGGATTTGGCCGTATTGGGCG (Integrated DNA Technologies). ZIKV RNA levels were interpolated against standard curves prepared by diluting RNA from uninfected tissue spiked with a known quantity of ZIKV (as determined by plaque assay) and expressed as PFU-equivalent per gram of tissue.

Blood was collected from infected mice, allowed to clot at 37°C and serum was separated by centrifugation at 10600*g* for 10 min and stored at -80°C. Total RNA in 50 μL serum was purified using QIAmp Viral RNA Mini Kit (QIAGEN). ZIKV RNA levels were determined by TaqMan Fast Virus 1-step Master Mix (Applied Biosystems), interpolated against a standard curve prepared using known quantities of ZIKV and expressed as PFU-equivalent per mL of serum.

### Histology and immunohistochemistry staining

Tissues were fixed in 10% neutral buffered formalin, embedded in paraffin, sectioned, and stained with hematoxylin and eosin. For immunohistochemistry staining of the ZIKV, 5-micron sections of paraffin-embedded tissues were washed with several changes of xylene to remove paraffin, and rehydrated through decreasing grades of ethanol (absolute to 70%) followed by deionized water. Endogenous alkaline phosphatase was blocked using Bloxall (Vector Labs) for 10 min. Tissues were washed with PBS containing 0.1% Tween 20, blocked with 5% normal mouse serum (Jackson Immunoresearch) for 30 min, and then incubated with the primary Ab (Zika virus Envelop protein antibody, GeneTex) diluted in PBS with 0.1% Tween 20 and 5% normal mouse serum for 2 h at room temperature. After washing, tissues were incubated with anti-rabbit IgG alkaline phosphatase (Vector Labs) for 30 min, then developed using Vector Red alkaline phosphatase substrate (Vector Labs), and counterstained with hematoxylin (Fisher). Slides were imaged using a Nikon Eclipse Ci microscope and Nikon SPOT 2 digital camera. The primary Ab used to stain for ZIKV envelop protein was validated by specific staining of ZIKV-infected Vero cells (S2 Fig). Uninfected and ZIKV-infected Vero cells processed as cell pellets, embedded in paraffin, and stained with the identical reagents as were the tissues were used as negative and positive controls, respectively, and were included in each staining protocol. Tissues from uninfected mice were also used as negative controls. Histology slides were reviewed by a board certified veterinary pathologist.

### Statistics

Statistical analyses were performed using Prism 5 (GraphPad Software). ZIKV RNA data were compared by nonparametric Mann-Whitney tests. ZIKV RNA levels that fell below the detection limit of our assays were arbitrarily assigned values 0.2 log below the limit of detection for nonparametric statistical analyses. Contingency data were analyzed by Fisher’s exact test using numbers in each group. Association/dissociation was analyzed by chi-square test. Weight data were analyzed by Student *t* test. *p<0.5, **p<0.01, ***p<0.001, ****p<0.0001; ns, not significant.

## Supporting information

S1 Fig ZIKV infection of pregnant C57BL/6J mice results in fetal growth restriction and reduced weight. Pregnant dams at E9.5 were left uninfected (uninfected), inoculated with Vero cell culture supernatant (Mock), 3.4 × 10^5^ PFU-equivalent of heat-inactivated ZIKV (Inact. ZIKV), or infected with 3.4 × 10^5^ PFU of ZIKV as described in Fig 1. Mice were sacrificed at 8 dpi. (**A**) Representative images of E17.5 embryos. The remaining embryos carried by ZIKV-infected dams exhibited growth restriction or were completely resorbed (placental residues). (**B**) Embryonic weight. The percentage indicates the embryos that had undergone complete resorption. Data shown are median with interquartile range. (ns, not significant; * p<0.05; **** p<0.0001 compared with uninfected group by Mann-Whitney test). Data are pooled from 5 independent experiments.
(TIF)

S2 Fig Validation of ZIKV envelope protein staining. Vero cells were grown to near confluency and infected with ZIKV at MOI of 0.1. Uninfected and ZIKV-infected Vero cells were harvested 3 days after infection and pelleted by centrifugation at 3800*g* for 15 min at 4°C. Media was aspirated and neutral buffered formalin was gently added to the cell pellet and allowed to fix for at least 24 h. After fixation, the uninfected and infected cell pellets were gently removed from fixative and processed alongside tissue samples into paraffin wax. Five micron sections were cut from each pellet. These prepared slides were stained with the identical reagents as were the tissues, and used as negative (uninfected) and positive (infected) controls, which were included in each staining protocol. ZIKV envelop protein (red) was detected in infected but not uninfected Vero cells. Scale bar, 25 μm.
(TIF)
