# Supplementary figures and images for: Zika virus infection in immunocompetent pregnant mice causes fetal damage and placental pathology in the absence of fetal infection
Source: PLoS Pathog. 2018 Apr 10;14(4):e1006994. doi: 10.1371/journal.ppat.1006994 (PMC5909921; doi:10.1371/journal.ppat.1006994)

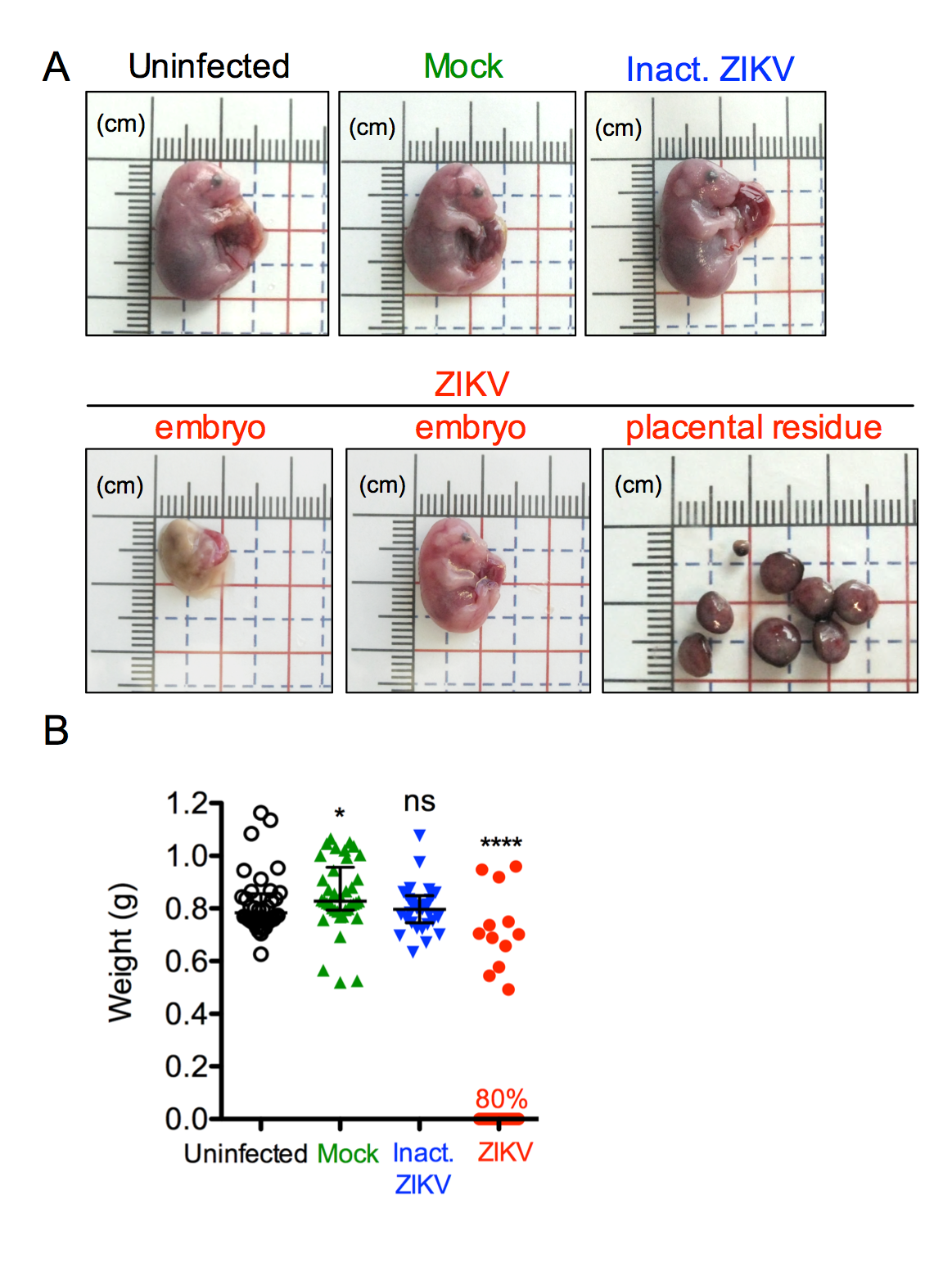

Supplement: S1 Fig — Pregnant dams at E9.5 were left uninfected (uninfected), inoculated with Vero cell culture supernatant (Mock), 3.4 × 10^5^ PFU-equivalent of heat-inactivated ZIKV (Inact. ZIKV), or infected with 3.4 × 10^5^ PFU of ZIKV as described in Fig 1. Mice were sacrificed at 8 dpi. (A) Representative images of E17.5 embryos. The remaining embryos carried by ZIKV-infected dams exhibited growth restriction or were completely resorbed (placental residues). (B) Embryonic weight. The percentage indicates the embryos that had undergone complete resorption. Data shown are median with interquartile range. (ns, not significant; * p<0.05; **** p<0.0001 compared with uninfected group by Mann-Whitney test). Data are pooled from 5 independent experiments. (TIF) [file ppat.1006994.s001.tif]

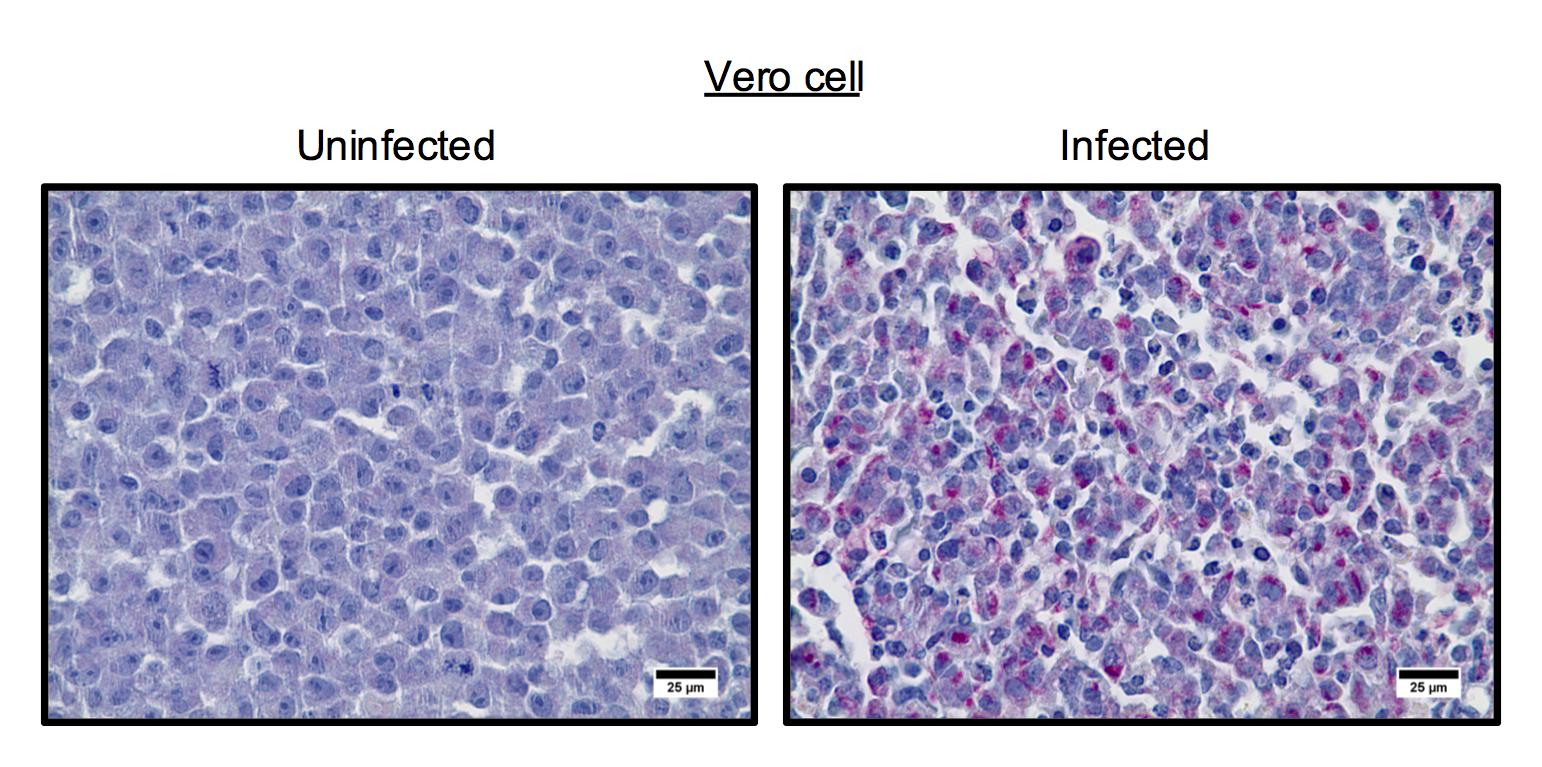

Supplement: S2 Fig — Vero cells were grown to near confluency and infected with ZIKV at MOI of 0.1. Uninfected and ZIKV-infected Vero cells were harvested 3 days after infection and pelleted by centrifugation at 3800g for 15 min at 4°C. Media was aspirated and neutral buffered formalin was gently added to the cell pellet and allowed to fix for at least 24 h. After fixation, the uninfected and infected cell pellets were gently removed from fixative and processed alongside tissue samples into paraffin wax. Five micron sections were cut from each pellet. These prepared slides were stained with the identical reagents as were the tissues, and used as negative (uninfected) and positive (infected) controls, which were included in each staining protocol. ZIKV envelop protein (red) was detected in infected but not uninfected Vero cells. Scale bar, 25 μm. (TIF) [file ppat.1006994.s002.tif]
